# Influence of high-temperature stress on rice growth and development. A review

**DOI:** 10.1016/j.heliyon.2022.e12651

**Published:** 2022-12-24

**Authors:** Sabin Shrestha, Janaki Mahat, Jenish Shrestha, Madhav K.C., Krishna Paudel

**Affiliations:** Institute of Agriculture and Animal Sciences, Tribhuvan University, Nepal

**Keywords:** Spikelet sterility, Pollination, Fertilization, Climate change, Global rice production, Low yield

## Abstract

High-temperature stress (HS) has become an alarming threat to the global food system. Rice, an important crop that supports almost half of the global population, is vulnerable to heat stress. Under the influence of HS, it shows various physiological and morphological symptoms that increase spikelet sterility, reduce grain yield, and even cause total crop failure. HS affects growth and yield in two ways: hindrance in the process of pollination and fertilization and reduction of the grain weight. The former is caused by (i) distortion of floral organs, (ii) tapetum degeneration, (iii) low pollen protein concentration, (iv) decline in pollen viability, (v) reduction in dehiscence of anther, (vi) low pollen dispersal, (vii) decrease in number of pollens on stigma, (viii) reduction in pollen grain germination, (ix) hindrance in extension of pollen tubes, and (x) shrinkage of stigma which ultimately cause spikelet infertility. The latter is caused by (i)reduced photosynthetic rate, (ii) a boost in senescence of functional leaves, (iii) reduction of biological synthesis of starch, (iv)reduced starch augmentation, (v) shrunk duration of grain filling, and (vi) declined grain weight which ultimately reduce the grain yield. However, some agronomic and breeding approaches have been adopted for developing thermo-resistant cultivars but the success is limited. In this paper, we have summarized the the morpho-physiological and molecular response of plant to HS, and a few possible management strategies.

## 1 Introduction

Climate change, one of the burning issues today, has been changing the earth's ecosystems more rapidly than ever. Since the 19^th^ century, human activities have increased the global temperature by 0.9 °C, particularly because of the emission of greenhouse gas (GHG) into the atmosphere (Arora, 2019). The environmental temperature largely regulates the seasonal growth and geographic distribution of crops (Li et al., 2018). In the 20^th^ century, global warming has resulted in a 0.5ᵒC rise in air temperature; in the 21^st^ century, the temperature is estimated to increase by 1.5–4.5ᵒC (Peraudeau et al., 2015) and high temperatures will become more usual in the coming days.

For efficient physiological processes such as growth, development, and reproduction, every crop has an ideal temperature range. Plant performance will be adversely affected by temperature above or below that range, resulting in a loss of yield potential. Rapidly warming climate causes high-temperature stress (HS) − warming above a threshold level for a certain period – resulting in irreversible harm to the development of plants (morphological and physiological) (Khan et al., 2019). It has been proposed that an inverse relation exists between global agricultural production and rising temperature, especially in the contexts of maize, wheat, rice, and barley (Karunaratne and Wheeler, 2015; Li et al., 2016; Yu et al., 2012).

Rice, an important crop worldwide, supports about half of the global population for their dietary requirements with 782 million tons of annual grain production being cultivated in 167 million hectares of area among 118 countries (Bagirov et al., 2020). In Southeast Asia, it provides more than 3/4^th^ of the caloric intake (Fitzgerald et al., 2009). Rice productivity in different geographical zones is challenged by frequent extreme weather events (Arshad et al., 2017). Temperature above 33ᵒC has led to the production decrement of rice in many places in the world (Arshad et al., 2017). The impact of HS on plant performance is mostly related to the strength, period, and timing (relative to the growth stages) of the stress, reproductive and grain-filling stages being the most critical phase (Fahad et al., 2017a). HS increases infertility of rice spikelet and it can even result in no yield (Satake and Yoshida, 1978; Fu et al., 2012), which can be attributed to restriction of pollen sterility, anther dehiscence, and unsuccessful germination on the stigma (Jagadish et al., 2010, 2013; Peraudeau et al., 2015). Under HS, lack of pollen tube extension in the carpel is the next key cause that leads to failure of pollination (Karapanos et al., 2010; Snider et al., 2011).

Considering the essence of rice in world food security and the impact of global warming on its seasonal growth and maturation, there is an exigent necessity to breed heat-tolerant varieties. Analysis of the biochemical and physiological features of rice that respond to HS, the genes as well as proteins entailed in high-temperature tolerance, and fundamental mechanisms behind the stress response are important steps in breeding heat-tolerant varieties (Janni et al., 2020; Raza et al., 2020; Sailaja et al., 2015a). In this paper, we explicate the influence of HS on rice performance along with the possible approaches toward the solution for enhancing yield potential.

## 2 Discussion

### 2.1 HS impact on the vegetative stage

Seed quality, dormancy, germination, and emergence, as well as seedling vigor and establishment, are all affected by HS throughout seed development (Brunei-Muguet et al., 2015; Finkelstein et al., 2008; Khan, 1976; Liu et al., 2019). HS has a significant detrimental impact on seed germination potential, resulting in lower seed viability and poor germination (Fahad et al., 2017a). Reduction in thermostability of the plasma membrane and membrane fluidity have been linked to decrease in germination and seed vigor caused by HS (Fahad et al., 2017b; Saidi et al., 2010) delaying Ca2+ signaling, kinases, and heat shock factors activation (Saidi et al., 2010; Sangwan et al., 2002). HS (35 °C) considerably reduced the overall seed size at maturity due to lower seed length, breadth, and weight in mature seeds during early seed development; at 39 °C, endosperm collapsed and seed viability get reduced significantly (Begcy et al., 2018).

**Figure 1:**
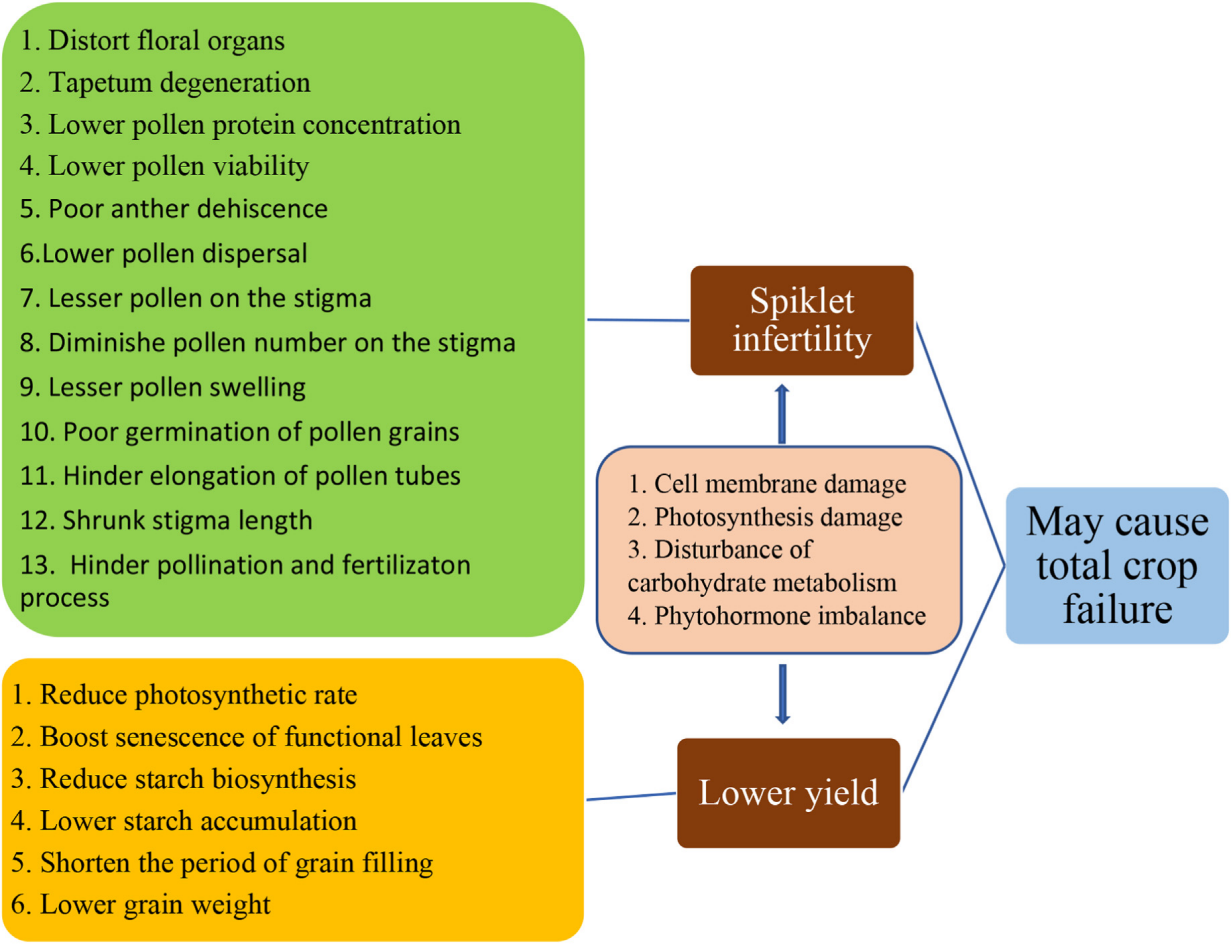
Influence of HS on morpho-physiology of rice.

When the temperature rises over 25–28 °C (the ideal temperature), the evapotranspiration rate rises, resulting in withering, curling, and yellowing of leaves, slow seedling and root growth, and potentially seedling death; in tillering stage, tiller number and biomass decline when exposed to high temperature (Liu et al., 2018; Xu et al., 2020) (see Figure 1). Rice seedling resistance to HS differs based on genetic makeup; HS has a greater influence on tiller and panicle number in japonica rice than in Indica rice (Wang et al., 2016). In the case of heat-resistance, Indica-japonica hybrid rice varieties demonstrate the highest level, ensued by Indica and japonica varieties (Prasanth et al., 2017).

### 2.2 HS impact on the reproductive stage

In rice, the reproductive phase – which begins with panicle initiation and continues up to physiological grain maturity – is the most sensitive phage to abiotic stresses (FU et al., 2008). Under HS (40 °C day/35 °C night) at the pre-flowering stage for 15 days, panicle number was reduced to 75% and overall rice yield per plant was lowered to 14%, compared to normal conditions i.e. 28 °C (Soda et al., 2018). The knowledge of the influence of HS on different reproductive phages is discussed below:

#### 2.2.1 Spikelet initiation

(Xu et al., 2020) stated, HS causes the floral organs to distort, diminishing their size and number. When exposed to HS (40ᵒC day/35ᵒC night), in India cultivar IR64, the spikelet number was reduced to 33% compared to normal growth conditions of 28ᵒC (Soda et al., 2018). HS also limits pollen grain expansion at the heading phase. A few hours of stress during flowering can reduce floral reproduction: embryo abortion (Matsui et al., 2000).

#### 2.2.2 Gametophyte and pollen development

Pollen viability and anther formation are more vulnerable to the warmth inside the floret than in the ovule (Fahad et al., 2017a). In different rice genotypes, HS causes both quantitative and qualitative changes in pollen proteins, which might result in pollen viability reduction and spikelet sterility (Das et al., 2014). The moisture content of pollen grains, critical for both pollen grain formation and dispersion, varies during landing on a well-suited stigma depending on environmental variations (Das et al., 2014). HS also declines iron uptake by pollen tubes or microspores which ultimately lowers the viability and germination of pollen (Jagadish et al., 2010). Poor pollen development may aggravates spikelet sterility (Jagadish et al., 2010). The initial microspore stage after meiosis was the most vulnerable to HS, and after seven days of HS (39 °C day/30 °C night), spikelet fertility was destroyed (Endo et al., 2009). HS in anther formation, especially at the pollen mother cell meiosis stage, can cause premature tapetal cell degeneration and fragmentation, affecting microspore nutrition and pollen wall construction, and induce pollen-grain abortion (G. Liu et al., 2020). HS causes tapetum disintegration and pollen sterility in rice, notably in the nascent anthers during the microspore phase (Endo et al., 2009). When exposed to HS (40 °C Day/35 °C night) for 10 days at the pollen mother cell meiosis stage, pollen viability and seed setting rate were lowered to 21.2 percent and 51.5 percent respectively when compared to normal conditions (30 °C Day/24 °C night).

#### 2.2.3 Anthesis

HS during anthesis inhibits the dehiscence of anther substantially (Arshad et al., 2017). Under HS, anthesis is the most crucial phase in rice (Jagadish et al., 2007; Prasad et al., 2006). HS, during anthesis, alters anther shape (Jagadish et al., 2010), decreases anther dehiscence (Tazib et al., 2015), lowers pollen viability (Endo et al., 2009), diminishes pollen number on stigma (Fu et al., 2016), lessens pollen swelling, reduces pollen germination on the stigma (Endo et al., 2009; Zhu et al., 2017), hinders lengthening of pollen tubes (Xu et al., 2020; Zhang et al., 2018a), and shrinks stigma length (Jagadish et al., 2010), all of which seriously interrupts the pollination and fertilization process, waning spikelet fertility (Shi et al., 2018). Spikelet sterility of rice was increased by as much as 80% when HS occurred at the time of the reproductive stage (Xu et al., 2020). Poor seed set is also caused by HS, which suppresses rice seed set shortly before or during anthesis (Prasad et al., 2006). Temperatures exceeding 35 °C for 5 days during anthesis causes sterile spikelets and, in extreme cases, complete seed loss (Jagadish et al., 2007); susceptibility to HS depends on one's genetic base. And, because of their varying organ temperatures, the quantity of grains on superior spikelet stigmas are more susceptible to HS than that on inferior spikelet (Fu et al., 2016).

#### 2.2.4 Pollination

Pollination and fertilization, which entails the splitting of stomium and anther dehiscence ensued by the release, deposition, and germination of pollen grains on stigma and polarized growth of pollen tubes, are all connected with spikelet fertility in rice (Wu et al., 2019a, Wu et al., 2019b). HS interferes with pollen germination and tube development by disrupting ion balance (such as Ca2+), carbohydrate metabolism (Firon et al., 2006), and pollen grains’ phytohormone concentration (Yan et al., 2002). HS at the flowering phase inhibits dehiscence of the anther, pollen germination on the pollen receptive tip of the pistil, and extension of the pollen tube in the pistil, resulting in poor pollination and fertilization and eventually spikelet sterility (Coast et al., 2016; Jiang et al., 2015; Snider et al., 2011; Fábián et al., 2019; Feng et al., 2018; Shi et al., 2018; C. Zhang et al., 2018b). Indehiscent anthers, caused by HS, are trapped inside the locules due to anther dehydration disruption (Wu et al., 2019a, Wu et al., 2019b). Pollen grains are well known for their inability to escape indehiscent anthers caused by HS. The principal cause of spikelet sterility in heat-stressed plants is inadequate shedding of pollen onto the pollen receptive tip of the pistil due to restricted anther dehiscence (Wang et al., 2019; Weerakoon et al., 2008). To assure more than 10 germinated pollens, over 20 pollen grains must be deposited on a stigma (Kobayashi et al., 2011). HS interrupts the shedding of pollen from dehiscent as well as indehiscent anthers.

#### 2.2.5 Spikelet sterility

HS during the flowering phase of rice plants severely decreases spikelet fertility, with the magnitude of the effect depending on genotypes and HS period (Zhang et al., 2018a).

##### 2.2.5.1 Decline in spikelet number

High temperature inhibits spikelet differentiation which worsen spikelet degeneration and lowers the number of spikelet (Jagadish et al., 2013; Wang et al., 2016). (Wu et al., 2017) discovered that high temperature prevents the development of spikelet which is linked to cytokine generation and breakdown. Furthermore, HS causes peroxide to accumulate in the spikelet, destroying the cellular structure and reducing spikelet quantity (Fu et al., 2015). HS also reduces pollen production by preventing anther filling during the panicle initiation phase (Wang et al., 2016).

##### 2.2.5.2 Inhibition of spikelet fertilization

HS causes spikelet infertility by reducing the viability of pollen, limiting dehiscence of the anther, and precluding pollen tube germination (Cao et al., 2015; Coast et al., 2016; Das et al., 2014; C. Zhang et al., 2018b). Under HS, poor dehiscence of anther and low counts of pollen grain on the stigma are the key aspects that reduce spikelet fertility (Kobayashi et al., 2011; Matsui and Omasa, 2002; Zhao et al., 2016). The activity of pollen is reduced in increased temperature due to stunting of pollen mother cell growth and aberrant tapetum disintegration (Abiko et al., 2005; DENG et al., 2010; Oshino et al., 2007). (Endo et al., 2009) discovered that temperatures as high as 39 °C cause poor nutrient storage in pollen grains, resulting in low pollen activity; nevertheless, normal pollen structure is restored in response to peroxide accumulation and decrease in carbon-carbon metabolism. The fertility rate of spikelet is primarily determined by anther dehiscence and the quantity of pollen grains on the stigma, although it has no bearing on pollen activity in HS conditions. HS have a stronger impact on pollen activity before spikelet flowering, which explains why spikelet exposed to high temperatures have a considerable relationship between fertilization rate and pollen viability (Wang et al., 2019).

Similarly (Cao et al., 2015) found that HS prevent carbohydrates from being transported to pollen, preventing pollen filling and lowering activity levels. The quantity of anther cell layers is inversely proportional to anther dehiscence (Matsui and Omasa, 2002). The viability of the rice stigma was assumed to be unaffected by HS (Satake and Yoshida, 1978), but (Wu et al., 2019) found that stigma viability decreases to 65-30% under 37 °C HS treatment. When subjected to high temperatures for 10 min with an open spikelet, the osmotic regulation of pollen was disrupted, causing a reduction in carbohydrate, vitamin C levels, and protein which inhibits pollen tube extension in stigma (Coast et al., 2016; Rieu et al., 2017). According to (Zhang et al., 2018b), poor pollen tube elongation is caused by a lack of indole-3-acetic acid in the stigma due to HS throughout the flowering stage.

### 2.3 Grain filling stage

HS has been demonstrated to reduce the weight of grains during grain filling (Dou et al., 2017). Affected plants' lower grain weight induced at grain filling stage has been ascribed to shorter duration of grain filling stage, varied grain filling rate and decrease in grain width (Cao et al., 2016; Wu et al., 2016). Heat treatment hinders assimilate production by reducing photosynthetic rate (ZHANG et al., 2009) and boosting senescence of functional leaves, which then lessens assimilate transfer to grains; warming may also impede early embryo (Cao et al., 2016) and seed development (Huang et al., 2019). The function of starch producing enzymes is frequently disturbed by HS (Cao et al., 2015). Moderately high temperatures enhance grain filling rate (Dou et al., 2017), but extremely high temperatures lower it; yet, the grain filling time is shortened at moderate and extremely high temperatures, lowering the ultimate yield. Decreased grain weight linked with declined nonstructural carbohydrates (Cao et al., 2016), undeveloped vascular bundles and reduced glume size has been attributed to the lower yield induced due to HS at the panicle initiation stage (ZHANG et al., 2009). Heat treatment at panicle commencement and grain filling lower grain weight (Wu et al., 2021).

### 2.4 Physiological impact of HS

#### 2.4.1 Membrane damage

Bio-membrane is regarded as the plant cell's most heat-sensitive element (Niu and Xiang, 2018; Sita et al., 2017a, Sita et al., 2017b). Elevated temperature can compromise plasma membrane form and function, change the proportion of saturated to unsaturated fatty acids, and cause protein denaturation, resulting in increased fluidity and permeability, affected membrane integrity, and enhanced organic and inorganic ion leakage from cells (Higashi and Saito, 2019; Niu and Xiang, 2018; Sita et al., 2017a, Sita et al., 2017b; Zhu et al., 2017).

#### 2.4.2 Photosynthesis damage

HS adversely affect photosynthesis; the most sensitive component is photosystem II (Wang et al., 2017); it destroys permeability of thylakoid membrane and even thylakoid grana disintegration and reduces chlorophyll content, and alters the photochemical reactions along with reducing the variable fluorescence to maximum fluorescence (Fv/Fm) ratio and photosynthetic rate (Hüve et al., 2011; Sailaja et al., 2015b; Q. L. Wang et al., 2018). As a result of the inactivation of Rubisco activase, it reduces the activity of ribulose-1,5-bisphosphate carboxylase/oxygenase (Rubisco) (Perdomo et al., 2017).

#### 2.4.3 Disturbance of carbohydrate metabolism and partitioning

In plants, HS disrupts glucose metabolism and photo-assimilate partitioning (Arshad et al., 2017; Bahuguna et al., 2017); it also disbalances phytohormones in the body. HS during the anthesis of rice results in discoloration of the sugar content in anthers that disturbs the normal nutrition supply for pollen development (Storme and Geelen, 2014; Rezaul et al., 2019). Under HS, vulnerable rice cultivars have high expression of the Carbon Starved Anthers (CSA) gene, whereas resistant cultivars have strong expression of the sugar transporter gene MST8 and cell wall invertase gene INV4, indicating sugar starvation as a role in spikelet sterility (Li et al., 2015).

#### 2.4.4 Phytohormone imbalance

In rice spikelet and developing kernel, HS reduces the level of active regulatory hormones viz. cytokinin, indole-3-acetic acid, gibberellin, and affects cell proliferation and panicle formation while also lowering spikelet quantity, pollen fertility, and kernel weight (Wu et al., 2016) but it increases the level of abscisic acid in anthers and seeds, leading to inhibition of the germination, seedling establishment and pollen abortion (Liu et al., 2019; Tang et al., 2007).

### 2.5 Molecular mechanisms of plant responses to heat stress

**Figure 2:**
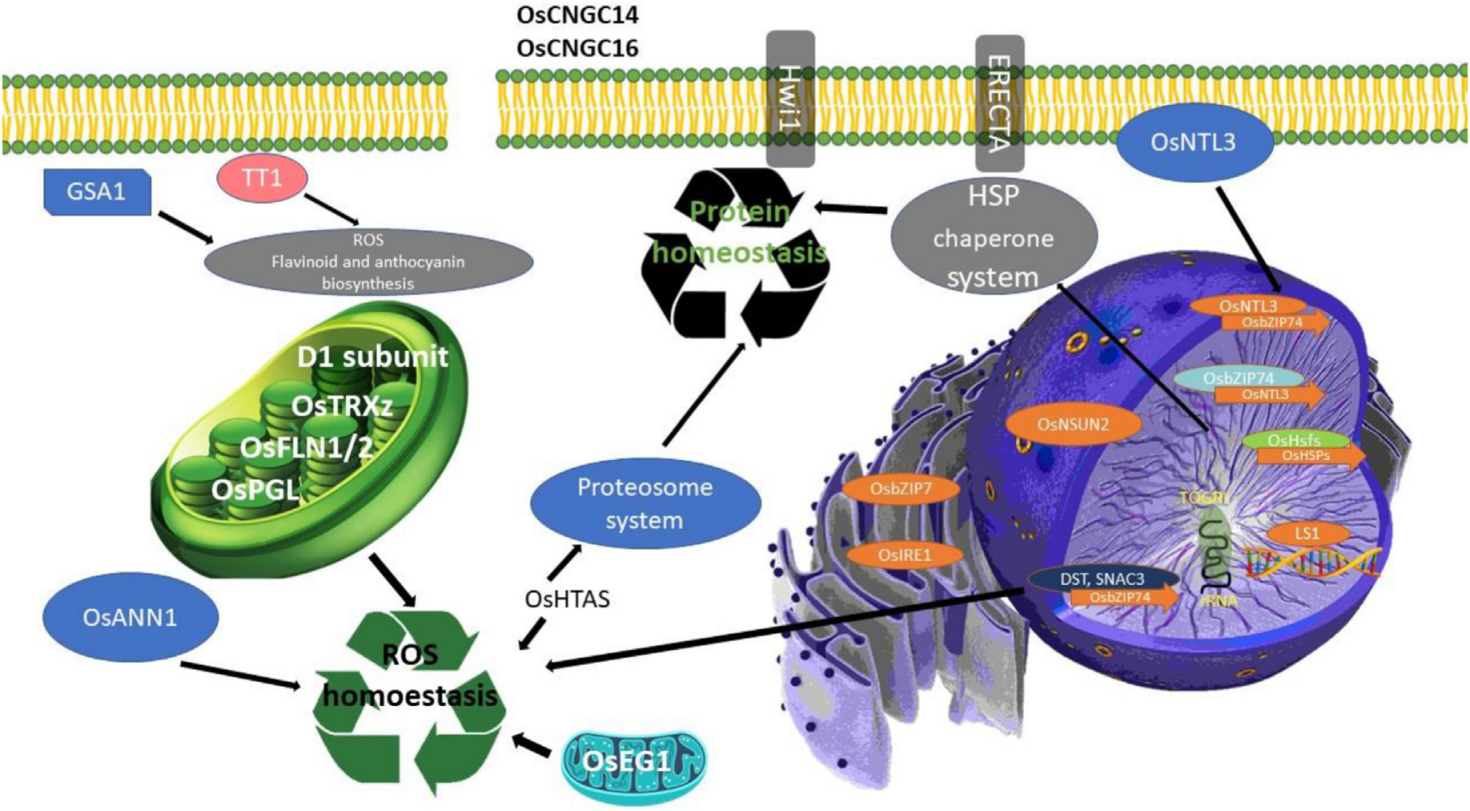
Molecular response of rice to HS.

Although mild increase in temperature do not cause extensive cell damage, they can alter morphogenesis (Li et al., 2015), biorhythms (Hsu and Harmer, 2014), and immunity response (Liu et al., 2015). HS can damage the membrane, burst ROS (reactive oxygen species), denature the cytotoxic proteins, and ultimately kill the plant (Zhang et al., 2019) (see Figure 2). Plants have developed various thermal responses to cope with heat stress. High temperature changes the fluidity of the membrane which not only changes the lipid composition and membrane lipid saturation level but also generates ROS (reactive oxygen species). Directly or indirectly, it activates the membrane-localized calcium channels, ensued by an increase in cytoplasmic Ca level, which play role in the activation or repression of activities related to the Ca2+/CaM-related kinases, phosphatases, and transcription factors (Kan and Lin, 2021).

Triggering the Ca signals, OsCNGC14 and OsCNGC16 respond to heat stress (Cui et al., 2020). In rice, the thermal response is regulated by leucine-repeat receptor-like kinases, ERECTA and Hwi1, through an unknown transduction pathway (Kan and Lin, 2021). Under HS, Endoplasmic Reticulum-localized OsbZIP74 moves to the nucleus and activates the expression of OsNTL3 ensued by the movement of membrane-localized OsNTL3 to the nucleus and regulation of expression of OsZIP74 (X. H. Liu et al., 2020; Lu et al., 2012). The restoration of damaged Photosystem II and PGL (pale green leaf, encoding chlorophyllide and oxygenase 1) in the chloroplast requires de novo synthesis of D1 subunit (Kan and Lin, 2021); the OsTRXz-OsFLN1/2 complex protects chloroplasts against heat-induced damage (Lv et al., 2017) whereas Mitochondria-localized EG1 protects the organelle and sustains floral robustness (Zhang et al., 2016).

Several genes cooperate to maintain ROS homeostasis such as OsANN1, SNAC3, OsHTAS, DST, and some other chloroplast genes (Kan and Lin, 2021). OsANN1, a calcium-binding annexin, enhances heat stress tolerance by modulating antioxidant accumulation (Qiao et al., 2015); DST (drought and salt tolerance), a C_2_H_2_ zinc finger transcription factor, controls the expression of genes that regulate H_2_O_2_-homeostasis and determines thermotolerance (Huang et al., 2009; J. Zhang et al., 2019). SNAC3, a NAC transcription factor, sustain ROS homeostasis and confer rice thermotolerance by activating many genes encoding ROS scavengers directly (Fang et al., 2015), and OsHTAS, a ubiquitin E3 ligase, promotes thermotolerance by controlling hydrogen peroxide buildup to change stomatal aperture in an ABA-dependent and DST-mediated process; it also aids in the clearance of unfolded proteins by interacting with ubiquitin/26S proteasome system components (Liu et al., 2015).

When protein homeostasis is interrupted, OsHSPs stabilize, renature, and help in the degradation of unfolded proteins, TT1 (THERMOTOLERANCE1), and the 26S proteasome's a2 component (Li et al., 2015), and OsHATS protect cells against cytotoxic denatured proteins via the 26S proteasome system. During HS, to maintain RNA homeostasis, TOGR1, OsNSUN2, SLG1, and AET1 preserve RNA processing, modification, and stability, whereas, LS1 (local lesion 1) maintains genome stability against DNA damage for DNA homeostasis (Dong et al., 2020). AET1, encoding a tRNA^His^ guanylyltransferase, plays a vital role in mRNA translation. GSA1, encoding a UDP-glucosyltransferase, protect rice against heat damage by controlling the accumulation of flavonoid glycosides to protect rice against heat damage (Dong et al., 2020).

### 2.6 Management strategies

To withstand HS, cultivation practices viz. management of moisture and nutrient, adjustment of sowing dates, use of plant growth regulators, as well as the intensification of pollination and fertilization has been reported (Fahad et al., 2017; Y. Wang et al., 2019). Changing agricultural practices, such as sowing time or selecting early morning blooming cultivars, inducing acclimation with growth regulators and nutrients, breeding for genetically heat resistant cultivars, and developing genetic alteration might all be utilized to increase rice thermo-tolerance (Wu et al., 2020a, Wu et al., 2020b). Developing varieties in partnership with plant breeders, biologists, and agronomists may be a more effective way to address these issues (Khan et al., 2019).

## 3 Conclusion and future research thrusts

Heat stress has affected the lifecycle of rice mainly hindering the process of pollination and fertilization, disrupting the molecular metabolism, and shortening the grain filling stage; it has adversely influenced global rice production through an increase in spikelet sterility, reduction in grain yield, and, in severe condition, even nullification of the grain yield. The most important field for future study is the development of heat-tolerant varieties through the combined effort of plant breeders and agronomists who must overcome the yield losses. Heat tolerant varieties grown in Nepal are not studied well for their genetic characteristics. For future study on the genetic base and attributes of heat-tolerant cultivars, it is crucial to take into account disaster warning, cultivar selection, adjustment of sowing time, and culture technology.

## Declarations

### Author contribution statement

All authors listed have significantly contributed to the development and the writing of this article.

### Funding statement

This research did not receive any specific grant from funding agencies in the public, commercial, or not-for-profit sectors.

### Data availability statement

Data included in article/supp. material/referenced in article.

### Declaration of interest's statement

The authors declare no competing interests.

### Additional information

No additional information is available for this paper.
